# Strong Correlation between the Case Fatality Rate of COVID-19 and the rs6598045 Single Nucleotide Polymorphism (SNP) of the Interferon-Induced Transmembrane Protein 3 (*IFITM3*) Gene at the Population-Level

**DOI:** 10.3390/genes12010042

**Published:** 2020-12-30

**Authors:** Yong-Chan Kim, Byung-Hoon Jeong

**Affiliations:** 1 Korea Zoonosis Research Institute, Jeonbuk National University, Iksan 54531, Jeonbuk, Korea; kych@jbnu.ac.kr; 2 Department of Bioactive Material Sciences and Institute for Molecular Biology and Genetics, Jeonbuk National University, Jeonju 54896, Jeonbuk, Korea

**Keywords:** COVID-19, case fatality rate, SARS-CoV-2, *IFITM3*, *ACE2*, *TMPRSS2*, *IL6*, *LZTFL1*, *ABO*, SNP

## Abstract

Coronavirus disease 2019 (COVID-19) is a fatal pandemic disease that is caused by infection with severe acute respiratory syndrome coronavirus 2 (SARS-CoV-2). As of 13 December, 2020, over 70,000,000 cases and 1,500,000 deaths have been reported over a period of several months; however, the mechanism underlying the pathogenesis of COVID-19 has not been elucidated. To identify the novel risk genetic biomarker for COVID-19, we evaluated the correlation between the case fatality rate of COVID-19 and the genetic polymorphisms of several potential COVID-19-related genes, including interferon-induced transmembrane protein 3 (*IFITM3*), the angiotensin I converting enzyme 2 (*ACE2*) gene, transmembrane protease, serine 2 (*TMPRSS2*), interleukin 6 (*IL6*), leucine zipper transcription factor-like protein 1 (*LZTFL1*), and the *ABO* genes, in various ethnic groups. We obtained the number of COVID-19 cases and deaths from the World Health Organization (WHO) COVID-19 dashboard and calculated the case fatality rate of each ethnic group. In addition, we obtained the allele distribution of the polymorphisms of the *IFITM3, ACE2, TMPRSS2, IL6, LZTFL1,* and *ABO* genes from the 1000 Genomes Project and performed Log-linear regression analysis using SAS version 9.4. We found different COVID-19 case fatality rates in each ethnic group. Notably, we identified a strong correlation between the case fatality rate of COVID-19 and the allele frequency of the rs6598045 single nucleotide polymorphism (SNP) of the *IFITM3* gene. To the best of our knowledge, this report is the first to describe a strong correlation between the COVID-19 case fatality rate and the rs6598045 SNP of the *IFITM3* gene at the population-level.

## 1. Introduction

Coronavirus disease 2019 (COVID-19) is a fatal acute respiratory disease caused by infection with severe acute respiratory syndrome coronavirus 2 (SARS-CoV-2) [1,2]. COVID19-infected patients have high fever, dry cough, dyspnea, and pneumonia [3]. As of 13 December, 2020, over 70,000,000 cases and 1,500,000 deaths (case fatality rate, over 2%) have been reported to the World Health Organization (WHO) from the WHO COVID-19 dashboard. Since the global threat posed by COVID-19 has been increasing due to shortages of adequate medical resources, it is very important to investigate the information regarding the mechanisms underlying the pathogenesis of COVID-19.

Previous studies have reported that the angiotensin I converting enzyme 2 (*ACE2*) and transmembrane protease serine 2 (*TMPRSS2*) genes play a pivotal role in the entry of SARS-CoV-2 into host cells. ACE2 is a major receptor of the spike protein of SARS-CoV-2 and polymorphisms of the *ACE2* gene modulates the susceptibility of SARS-CoV-2 infection via elevation in expression level of ACE2. In addition, TMPRSS2 is a serine protease and plays a role in the spike protein priming of SARS-CoV-2 for viral invasion [4,5]. Thus, in a previous study, the genetic polymorphisms affecting the expression level of the *TMPRSS2* gene have been suggested as novel candidates in the severity of COVID-19 in Italy [6]. However, a previous study did not observe an association between the genetic polymorphisms of the *ACE2* and *TMPRSS2* genes and SARS-CoV-2 infection [7]. In addition, the rs12252 single nucleotide polymorphism (SNP) of the interferon-induced transmembrane protein 3 (*IFITM3*) gene is related to the severity of COVID-19 in the Han Chinese population [8]. Furthermore, since the rs180079 SNP of the interleukin 6 (*IL6*) gene has been affected by the severity of several types of lung diseases, including chronic obstructive pulmonary disease (COPD) and pneumonia, the *IL6* polymorphism is presumed to confer susceptibility to COVID-19 [9]. A recent genome-wide association study (GWAS) reported that two polymorphisms, the rs11385942 insertion/deletion polymorphism of the leucine zipper transcription factor-like protein 1 (*LZTFL1*) gene and the rs657152 SNP of the *ABO* gene, are related to severe COVID-19 cases with respiratory failure [10]. However, an association between genetic variants and the case fatality of COVID-19 has not been determined.

In the present study, to find the novel genetic biomarker for the severity of COVID-19, we evaluated a correlation at the population-level between the case fatality rate of COVID-19 and genetic polymorphisms of several potential COVID-19-related genes, including *IFITM3, ACE2, TMPRSS2, IL6, LZTFL1,* and the *ABO* genes. To investigate this correlation, we obtained the number of cases and deaths of COVID-19 from the WHO COVID-19 dashboard and calculated the case fatality rate of each ethnic group. In addition, we obtained allele frequencies of the polymorphisms of the *IFITM3, ACE2, TMPRSS2 IL6, LZTFL1,* and *ABO* genes from the 1000 Genomes Project and performed log-linear regression analysis at the population-level.

## 2. Results

### 2.1. Information on the Polymorphisms of COVID-19-Related Genes

We selected a total of 26 polymorphisms, which have been previously reported for relationship with SARS-CoV-2, influenza A H1N1 pandemic 2009 virus and COPD [6,8,9,10,11,12,13]. Three SNPs of the *IFITM3* gene, rs12252, rs34481144 and rs6598045, were analyzed in this study. Four SNPs of the *ACE2* gene, rs2285666, rs35803318, rs2074192 and rs2106809, were analyzed in this study. Fifteen SNPs of the *TMPRSS2* gene and 1 insertion/deletion polymorphism, that is, rs2070788, rs2298659, rs17854725, rs12329760, rs3787950, rs463727, rs9974589, rs34624090, rs7364083, rs55964536, rs734056, rs4290734, rs34783969, rs11702475, rs35899679, and rs35041537, were analyzed in this study. One SNP of the *IL6* gene, rs1800795, was analyzed in this study. One insertion/deletion polymorphism of the *LZTFL1* gene, rs11385942, was analyzed in this study. One SNP of the *ABO* gene, rs657152, was analyzed in this study. Detailed information regarding these genes is presented in Supplementary Table S1.

### 2.2. Ethnic Differences of Case Fatality Rates among COVID-19 Patients

We obtained the number of cases and deaths of COVID-19 patients according to their ethnic groups, including African, European, American, East Asian, and South Asian groups, from the WHO COVID-19 dashboard (as of 29 June, 2020). Detailed information on worldwide case fatality rates of COVID-19 was described in Table 1. Interestingly, the case fatality rate of COVID-19 varies according to ethnic background.

### 2.3. Regression Analysis between the Case Fatality Rate of COVID-19 and the Polymorphisms of COVID-19-Related Genes

**Figure 1:**
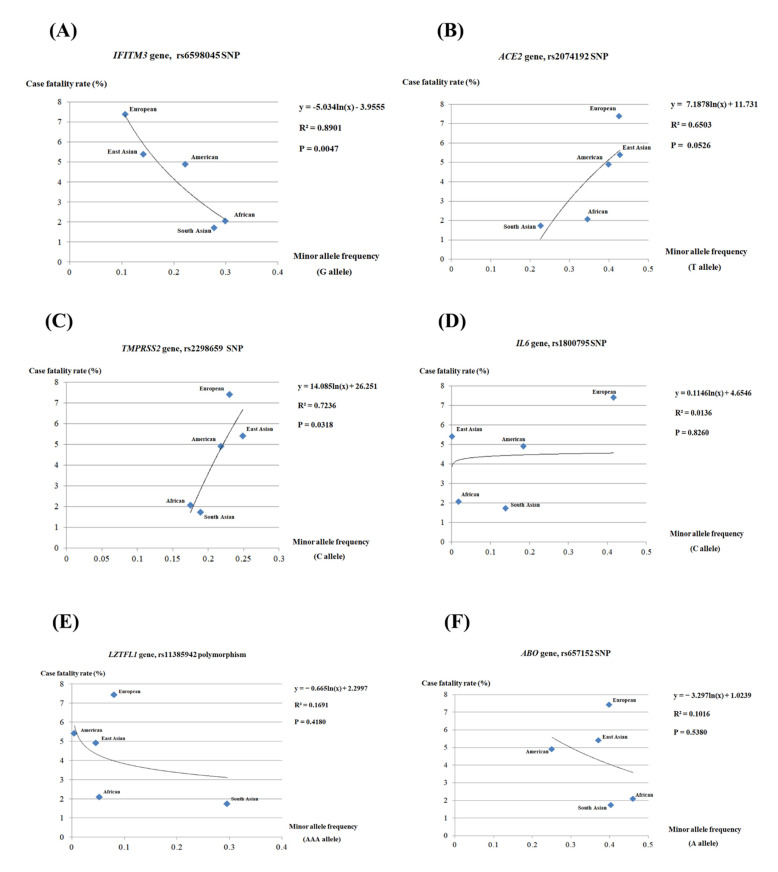
(**A**) Log-linear regression relationships between the case fatality rate of coronavirus disease-2019 (COVID-19) and minor allele frequency (MAF) of the rs6598045 single nucleotide polymorphism (SNP) of the interferon-induced transmembrane protein 3 (*IFITM3)* gene. (**B**) Log-linear regression relationships between the case fatality rate of COVID-19 and MAF of the rs2074192 SNP of the angiotensin I converting enzyme 2 (*ACE2*) gene. (**C**) Log-linear regression relationships between the case fatality rate of COVID-19 and MAF of the rs2298659 SNP of the transmembrane protease serine 2 (*TMPRSS2*) gene. (**D**) Log-linear regression relationships between the case fatality rate of COVID-19 and MAF of the rs1800795 SNP of the interleukin 6 (*IL6*) gene. (**E**) Log-linear regression relationships between the case fatality rate of COVID-19 and MAF of the rs11385942 insertion/deletion polymorphism of the leucine zipper transcription factor-like protein 1 (*LZTFL1*) gene. (**F**) Log-linear regression relationships between the case fatality rate of COVID-19 and MAF of the rs657152 SNP of the *ABO* gene.

To identify a correlation between the case fatality rate and the minor allele frequencies (MAFs) of the polymorphisms of the COVID-19-related genes, we performed log-linear regression analysis. Detailed values of *r*^2^ and *p*-values are presented in Supplementary Table S2. Notably, only the MAF of the rs6598045 SNP of the *IFITM3* gene showed a strong correlation (*r*^2^ = 0.8901, *p* = 0.0047) with the case fatality rate of COVID-19 (Figure 1A). Among the SNPs of the *ACE2* gene, the rs2074192 SNP showed the highest correlation (*r*^2^ = 0.6503, *p* = 0.0526) with the case fatality rate of COVID-19 (Figure 1B). Among the SNPs of the *TMPRSS2* gene, the rs2298659 SNP showed the strongest correlation (*r*^2^ = 0.7236, *p* = 0.0318) with the case fatality rate of COVID-19 (Figure 1C). In the *IL6* gene, the rs1800795 SNP showed a weak correlation (*r*^2^ = 0.0136, *p* = 0.8260) with the case fatality rate of COVID-19 (Figure 1D). In the *LZTFL1* gene, the rs11385942 polymorphism showed a weak correlation (*r*^2^ = 0.1691, *p* = 0.4180) with the case fatality rate of COVID-19 (Figure 1E). In the *ABO* gene, the rs657152 SNP showed a weak correlation (*r*^2^ = 0.1016, *p* = 0.5380) with the case fatality rate of COVID-19 (Figure 1F).

## 3. Discussion

In the present study, we identified a strong correlation between the case fatality rate of COVID-19 and the allele frequency of the rs6598045 SNP of the *IFITM3* gene. Previous studies have reported that the IFITM3 protein showed potent antiviral capacity to a wide range of viruses, including influenza A viruses (IAVs), Ebola virus (EBOV), Marburg virus (MARV), SARS-CoV, dengue virus (DEV), West Nile virus (WNV), Zika virus (ZIKV), and foot-and-mouth disease virus (FMDV) [14,15,16,17,18,19]. The IFITM3 protein has been presumed to physically inhibit the endocytosis of several viruses by constructing a chain-like structure on the cell membrane between IFITM3 protein monomers. Thus, polymorphisms that affect the function and expression level of the IFITM3 protein play a crucial role in the antiviral capacity of the IFITM3 protein. The rs12252 SNP, which is located on the splicing receptor, is related to the truncated form of the IFITM3 protein and is associated with the severity of the 2009 H1N1 influenza A pandemic and COVID-19 [13,20,21]. However, previous studies using RNA sequencing did not find a splicing form of the IFITM3 protein induced by the rs12252 SNP and the correlation between the rs12252 SNP and the severity of 2009 pandemic H1N1 influenza A in several ethnic groups [22,23]. The rs34481144 SNP is located on the regulatory region of the *IFITM3* gene and the binding site of CTCF. According to the allele of the rs34481144 SNP, transcriptional up/down regulation via binding affinity of the transcription factor of the *IFITM3* gene is modified and is associated with the severity of 2009 pandemic H1N1 influenza A [12,24]. However, East Asian showed a notably low MAF of the rs34481144 SNP (0.006), and the Korean population did not show polymorphisms in the rs34481144 SNP (0) [11]. In addition, the Korean population did not show association between the rs34481144 SNP and the susceptibility of 2009 pandemic H1N1 influenza A. The rs6598045 SNP, which is located on the proximal promoter of the *IFITM3* gene, is related to transcriptional efficiency via the binding ability of the transcription factor TFII-I and is a novel candidate SNP associated with the susceptibility to 2009 pandemic H1N1 influenza A infection [11]. Although viral receptor and binding protein are different between SARS-CoV-2 and 2009 pandemic H1N1 influenza A virus, both viruses showed several commonalities including RNA genome, identical target cell, similar respiratory symptoms and co-morbidities related to severe illness [25,26]. Thus, we selected polymorphisms, which have been previously reported for an association with SARS-CoV-2 and influenza A H1N1 pandemic 2009 virus [6,8,9,10,11,12,13].

In the present study, we performed regression analysis and found a strong correlation between the rs6598045 SNP and the case fatality rate of COVID-19 (Figure 1A, Supplementary Table S2). Interestingly, three SNPs of the *IFITM3* gene showed different *p*-values (Supplementary Table S2). Although those SNPs are located close within 1 kb (Supplementary Table S1), the mechanism related to innate immune response is very different among them. Thus, rs6598045 SNP seems likely to play a more dominant role in COVID-19 severity than other two SNPs. In addition, the rs2074192 SNP of the *ACE2* gene and rs2298659 SNP of the *TMPRSS2* gene also showed the highest correlation among other *ACE2* and *TMPRSS2* polymorphisms, respectively (Figure 1B,C, Supplementary Table S2). However, we carried out a simplified analysis with exclusion of various factors, including the medical environment, average age, and quarantine system. Notably, the saturation of the medical system and population age played a pivotal role in case fatality rate in COVID-19 [27]. In addition, active quarantine measure, including prompt implement of large-scale viral testing and restrictions on rallies and crowd gatherings contributed to a remarkable decline of the case fatality rate in the early days of the COVID-19 outbreak in the Republic of Korea [28]. Since medical environment, average age and quarantine system had an impact on case fatality of COVID-19 and were quite different among countries, it must be considered as a limitation in this study. Furthermore, since case fatality rate depends on the amount of testing conducted in a community, there is the possibility of bias. Although population-level regression analysis is a useful and convenient tool in a fast-moving pandemic for identifying the relationship between genetic factors and clinical outcomes, there is the possibility of ecological fallacy, which is an error of making conclusions about individuals through only interpretation of statistical data at the population-level [29,30]. Since not all of the genetic data of each country was available, we collected genetic data in five major groups from 1000 genome project. However, since these large multinational groups are very heterogenous populations, which contain diverse population substructure, different minor allele frequencies, and various case fatality rates via different level of COVID-19 control, there is limitation of this simplified population-level analysis. In addition, we only analyzed using minor allele frequency in the present study. However, the heterozygote and homozygote of genotype frequency may possibly have different effects, and further investigation based on the genotype frequency is highly desirable in the future [31]. Furthermore, to validate the findings of the current study, we will investigate the correlation of these SNPs with phenotype of COVID-19, including asymptomatic, mild, severe, and fatal cases using a case-control study at the individual level in the future. Finally, since our analysis was based on the data up to June 2020, there is a gap to reflect the fast-moving current status of COVID-19. However, because the data up to June 2020 reflected the initial situation in which there was not enough the global preparation for COVID-19, these data are also very meaningful. In recent studies, several GWAS studies have been carried out to identify novel genetic biomarkers for severity of COVID-19. One study has been performed in Italian and Spanish COVID-19 patients with respiratory failure. This study indicated that solute carrier family 6 member 20 gene *(SLC6A20), LZTFL1,* C-C chemokine receptor type 9 gene *(CCR9),* FYVE and coiled-coil domain autophagy adaptor 1 gene *(FYCO1),* C-X-C chemokine receptor type 6 gene *(CXCR6),* X-C Motif Chemokine Receptor 1 gene *(XCR1),* and *ABO* genes were involved in severity of COVID-19 [10]. The other study has been done in British COVID-19 patients admitted to the intensive care unit. This study showed that interferon α and β receptor subunit 2 gene *(IFNAR2),* tyrosine kinase 2 gene *(TYK2)* and chemokine receptor type 2 gene (*CCR2)* gene were associated with severity of COVID-19 [32]. Although the same genes were not identified as biomarkers in both studies, immune-related genes were commonly associated with severity of COVID-19. Since our study also suggested that *IFITM3* gene, downstream effector of innate immune system could also be involved in the case fatality rate of COVID-19, further GWAS analysis in death patients with COVID-19 in various ethnic groups is highly required to validate genetic biomarkers for the case fatality rate of COVID-19 in the future.

## 4. Materials and Methods

### 4.1. Data Collection

The number of confirmed cases and deaths of COVID-19 was obtained from the WHO COVID-19 dashboard. The worldwide distribution of the allele frequencies on polymorphisms of the *IFITM3, ACE2, TMPRSS2, IL6, and ABO* genes was obtained from the 1000 Genomes Project. The polymorphisms analyzed in this study were selected from polymorphisms, which have been reported for an association with SARS-CoV-2, influenza A H1N1 pandemic 2009 virus, and COPD in previous studies [6,8,9,10,11,12,13,33].

### 4.2. Statistical Analysis

All statistical analyses were performed using SAS version 9.4 (SAS Institute Inc., Cary, NC, USA). Case fatality rate was calculated as follows: case fatality rate (%) = (number of cases)/(number of deaths) × 100. The relationships between the case fatality rate of COVID-19 and the MAF of the polymorphisms of the COVID-19-related genes were evaluated using log-linear regression (Poisson model) analysis. The regression analysis was performed on each polymorphism for a total of 26 times. Adjusted *p*-values < 0.002 using Bonferroni correction were considered statistically significant.

## 5. Conclusions

In conclusion, we obtained the worldwide case fatality rates of COVID-19 and genetic information on the *IFITM3, ACE2, TMPRSS2,* and *IL6* genes. We performed log-linear regression analysis between the case fatality rate of COVID-19 and allele frequencies of the polymorphisms of the *IFITM3, ACE2, TMPRSS2,* and *IL6* genes in several ethnic groups. We identified a strong correlation between the case fatality rate of COVID-19 and the allele frequency of the rs6598045 SNP *IFITM3* gene. To the best of our knowledge, this report is the first to describe a strong correlation between COVID-19 and the rs6598045 SNP of the *IFITM3* gene at the population-level.

## Figures and Tables

**Table 1:** Information on cases and deaths of coronavirus disease 2019 (COVID-19) in several ethnic groups (29 June, 2020).

Subject Groups	Matching Groups in1000 Genome Project	Case	Death	Case Fatality Rate (%)
African ^a^	African (AFR)	278,815	5785	2.07
European ^b^	European (EUR)	2,656,437	196,541	7.40
American ^c^	American (AMR)	4,933,972	241,931	4.90
East Asian ^d^	East Asian (EAS)	104,035	5620	5.40
South Asian ^e^	South Asian (SAS)	336,933	5813	1.73

African ^a^: Algeria, Angola, Benin, Botswana, Burkina Faso, Burundi, Cabo Verde, Cameroon, Central African Republic, Chad, Comoros, Congo, Cote d’Ivoire, Democratic Republic of Congo, Equatorial Guinea, Eritrea, Eswatini, Ethiopia, Gabon, Gambia, Ghana, Guinea, Guinea-Bissau, Kenya, Lesotho, Liberia, Madagascar, Malawi, Mauritania, Mauritius, Mozambique, Namibia, Niger, Nigeria, Rwanda, Sao Tome and Principe, Senegal, Seychelles, Sierra Leone, South Africa, South Sudan, Togo, Uganda, United Republic of Tanzania, Zambia and Zimbabwe. European ^b^: Albania, Andorra, Armenia, Austria, Azerbaijan, Belarus, Belgium, Bosnia and Herzegovina, Bulgaria, Croatia, Cyprus, Czechia, Denmark, Estonia, Finland, France, Georgia, Germany, Greece, Hungary, Iceland, Ireland, Israel, Italy, Kazakhstan, Kyrgyzstan, Latvia, Lithuania, Luxembourg, Malta, Monaco, Montenegro, Netherlands, North Macedonia, Norway, Poland, Portugal, Republic of Moldova, Romania, Russian Federation, San Marino, Serbia, Slovakia, Slovenia, Spain, Sweden, Switzerland, Tajikistan, Turkey, Turkmenistan, Ukraine, United Kingdom of Great Britain, and Northern Ireland, and Uzbekistan. American ^c^: Antigua, Barbuda, Argentina, Bahamas, Barbados, Belize, Bolivia, Brazil, Canada, Chile, Colombia, Costa Rica, Cuba, Dominica, Dominican Republic, Ecuador, El Salvador, Grenada, Guatemala, Guyana, Haiti, Honduras, Jamaica, Mexico, Nicaragua, Panama, Paraguay, Peru, Saint Kitts and Nevis, Saint Lucia, Saint Vincent and the Grenadines, Suriname, Trinidad and Tobago, United States of America, Uruguay and Venezuela. East Asian ^d^: China, Japan; South Asian ^e^: Sri Lanka, Bangladesh, and India.

## Data Availability

All data generated or analyzed during this study are available from the corresponding author on reasonable request.

## Supplementary Materials

The following are available online at https://www.mdpi.com/2073-4425/12/1/42/s1, Supplementary Table S1: Detailed information on polymorphisms of the COVID-19-related genes analyzed in this study, Supplementary Table S2: Summary of correlation analysis between the case fatality rate of COVID-19 and allele frequencies of the polymorphisms of the *IFITM3, ACE2, TMPRSS2*, *IL6, LZTFL1, and ABO* genes in several ethnic groups.

## Abbreviations

SNP	Single nucleotide polymorphism
*IFITM3*	Interferon-induced transmembrane protein 3 gene
COVID-19	Coronavirus disease 2019
SARS-CoV-2	Severe acute respiratory syndrome coronavirus 2
*ACE2*	Angiotensin I converting enzyme 2 gene
*TMPRSS2*	Transmembrane protease, serine 2 gene
*IL6*	Interleukin 6 gene
*LZTFL1*	Leucine zipper transcription factor-like protein 1 gene
WHO	World Health Organization
COPD	Chronic obstructive pulmonary disease
GWAS	Genome-wide association study
MAF	Minor allele frequency
*SLC6A20*	Solute carrier family 6 member 20 gene
*CCR9*	C-C chemokine receptor type 9 gene
*FYCO1*	FYVE and coiled-coil domain autophagy adaptor 1 gene
*CXCR6*	C-X-C chemokine receptor type 6 gene
*XCR1*	X-C Motif Chemokine Receptor 1 gene
*IFNAR2*	Interferon α and β receptor subunit 2 gene
*TYK2*	Tyrosine kinase 2 gene
*CCR2*	Chemokine receptor type 2 gene
