# Supplementary material for: Strong Correlation between the Case Fatality Rate of COVID-19 and the rs6598045 Single Nucleotide Polymorphism (SNP) of the Interferon-Induced Transmembrane Protein 3 (IFITM3) Gene at the Population-Level
Source: Genes (Basel). 2020 Dec 30;12(1):42. doi: 10.3390/genes12010042 (PMC7824003; doi:10.3390/genes12010042)
Supplement: Supplementary file 1 [file genes-12-00042-s001.pdf]

Supplementary Table 1 Detailed information on polymorphisms of the COVID-19-related genes analyzed in this study.

Gene	Gene location	Exon count	Polymorphism	Position	Major allele	Minor allele	Classification
IFITM3	11p15.5	2	rs12252	NC_000011.10:g.320772A>G	A	G	Splice region variant
			rs34481144	NC_000011.10:g.320836C>T	C	T	5' UTR variant
			rs6598045	NC_000011.10:g.321001A>G	A	G	5' UTR variant
ACE2	Xp22.2	19	rs2285666	NC_000023.11:g.15592225C>T	C	T	Splice region variant
			rs35803318	NC_000023.10:g.15582209C>T	C	T	Synonymous variant
			rs2074192	NC_000023.11:g.15564667C>T	C	T	Intron variant
			rs2106809	NC_000023.11:g.15599938A>G	A	G	Intron variant
TMPRSS2	21q22.3	15	rs2070788,	NC_000021.9:g.41470061G>A	A	G	Intron variant
			rs2298659	NC_000021.9:g.41473447G>A	G	A	Synonymous variant
			rs17854725	NC_000021.9:g.41473456A>G	G	A	Synonymous variant
			rs12329760	NC_000021.9:g.41480570C>T	C	T	Missense variant
			rs3787950	NC_000021.9:g.41494369T>C	T	C	Synonymous variant
			rs463727	NC_000021.9:g.41464259T>A	T	A	3' UTR variant
			rs9974589	NC_000021.9:g.41470196A>C	A	C	Intron variant
			rs34624090	NC_000021.9:g.41471517dup	CCC	CCCC	Intron variant
			rs7364083	NC_000021.9:g.41472130G>A	G	A	Intron variant
			rs55964536	NC_000021.9:g.41473711C>T	C	T	Intron variant
			rs734056	NC_000021.9:g.41480393C>A	C	A	Intron variant
			rs4290734	NC_000021.9:g.41481156A>G	A	G	Intron variant
			rs34783969	NC_000021.9:g.41482711A>T	A	T	Intron variant
			rs11702475	NC_000021.9:g.41485641C>T	C	T	Intron variant
			rs35899679	NC_000021.9:g.41491393C>A	C	A	Intron variant
			rs35041537	NC_000021.9:g.41491442C>T	C	T	Intron variant
IL6	7p15.3	6	rs1800795	NC_000007.14:g.22727026C>G	C	G	Intron variant
LZTFL1	3p21.31	15	rs11385942	NC_000003.12:g.45834969dup,	AA	AAA	Non coding transcript exon variant
ABO	9q34.2,	7	rs657152	NC_000009.12:g.133263862A>C	C	A	Intron variant

Supplementary Table 2 Summary of correlation analysis between the case fatality rate of COVID-19 and allele frequencies of the polymorphisms of the *IFITM3, ACE2, TMPRSS2* and *IL6* genes in several ethnic groups.

Gene	Polymorphisms	Matching groups in1000 genome project	Allele frequencies		Case fatality rate (%)	r^2	P-value
IFITM3	rs12252		A	G		0.1725	0.0419
		African (AFR)	0.740	0.260	2.07		
		European (EUR)	0.959	0.041	7.40		
		American (AMR)	0.823	0.177	4.90		
		East Asian (EAS)	0.472	0.528	5.40		
		South Asian (SAS)	0.853	0.147	1.73		
	rs34481144		C	T		0.017	0.8055
		African (AFR)	0.957	0.043	2.07		
		European (EUR)	0.538	0.462	7.40		
		American (AMR)	0.767	0.233	4.90		
		East Asian (EAS)	0.994	0.006	5.40		
		South Asian (SAS)	0.793	0.207	1.73		
	rs6598045		A	G		0.8901	0.0047
		African (AFR)	0.701	0.299	2.07		
		European (EUR)	0.894	0.106	7.40		
		American (AMR)	0.778	0.222	4.90		
		East Asian (EAS)	0.859	0.141	5.40		
		South Asian (SAS)	0.723	0.277	1.73		
ACE2	rs2285666		C	T		0.0139	0.8243
		African (AFR)	0.789	0.211	2.07		
		European (EUR)	0.765	0.235	7.40		
		American (AMR)	0.664	0.336	4.90		
		East Asian (EAS)	0.463	0.537	5.40		
		South Asian (SAS)	0.521	0.479	1.73		
	rs35803318		C	T		0.3933	0.1826
		African (AFR)	0.999	0.001	2.07		
		European (EUR)	0.946	0.054	7.40		
		American (AMR)	0.929	0.071	4.90		
		East Asian (EAS)	1.000	0.000	5.40		
		South Asian (SAS)	1.000	0.000	1.73		

	rs2074192		C	T		0.6503	0.0526
		African (AFR)	0.654	0.346	2.07		
		European (EUR)	0.574	0.426	7.40		
		American (AMR)	0.601	0.399	4.90		
		East Asian (EAS)	0.572	0.428	5.40		
		South Asian (SAS)	0.774	0.226	1.73		
	rs2106809		A	G		0.0511	0.6666
		African (AFR)	0.911	0.089	2.07		
		European (EUR)	0.753	0.247	7.40		
		American (AMR)	0.676	0.324	4.90		
		East Asian (EAS)	0.478	0.522	5.40		
		South Asian (SAS)	0.517	0.483	1.73		
TMPRSS2	rs2070788		G	A		0.1475	0.4522
		African (AFR)	0.274	0.726	2.07		
		European (EUR)	0.464	0.534	7.40		
		American (AMR)	0.494	0.506	4.90		
		East Asian (EAS)	0.356	0.644	5.40		
		South Asian (SAS)	0.466	0.534	1.73		
	rs2298659		G	A		0.7236	0.0318
		African (AFR)	0.825	0.175	2.07		
		European (EUR)	0.770	0.230	7.40		
		American (AMR)	0.782	0.218	4.90		
		East Asian (EAS)	0.751	0.249	5.40		
		South Asian (SAS)	0.811	0.189	1.73		
	rs17854725		A	G		0.0021	0.9316
		African (AFR)	0.661	0.339	2.07		
		European (EUR)	0.458	0.542	7.40		
		American (AMR)	0.589	0.411	4.90		
		East Asian (EAS)	0.879	0.121	5.40		
		South Asian (SAS)	0.556	0.444	1.73		
	rs12329760		C	T			
		African (AFR)	0.713	0.287	2.07	0.0013	0.9451

	European (EUR)	0.764	0.236	7.40		
	American (AMR)	0.846	0.154	4.90		
	East Asian (EAS)	0.638	0.362	5.40		
	South Asian (SAS)	0.774	0.226	1.73		
rs3787950		T	C		0.6165	0.0643
	African (AFR)	0.782	0.218	2.07		
	European (EUR)	0.921	0.079	7.40		
	American (AMR)	0.939	0.061	4.90		
	East Asian (EAS)	0.845	0.155	5.40		
	South Asian (SAS)	0.743	0.257	1.73		
rs463727		T	A		0.0009	0.9547
	African (AFR)	0.921	0.079	2.07		
	European (EUR)	0.543	0.457	7.40		
	American (AMR)	0.728	0.272	4.90		
	East Asian (EAS)	0.994	0.006	5.40		
	South Asian (SAS)	0.645	0.355	1.73		
rs9974589		A	C		0.1217	0.3489
	African (AFR)	0.273	0.727	2.07		
	European (EUR)	0.464	0.536	7.40		
	American (AMR)	0.493	0.507	4.90		
	East Asian (EAS)	0.356	0.644	5.40		
	South Asian (SAS)	0.465	0.535	1.73		
rs34624090		CCC	CCCC		0.0063	0.8809
	African (AFR)	0.904	0.096	2.07		
	European (EUR)	0.558	0.442	7.40		
	American (AMR)	0.759	0.241	4.90		
	East Asian (EAS)	0.994	0.006	5.40		
	South Asian (SAS)	0.613	0.387	1.73		
rs7364083		G	A		0.2269	0.3396
	African (AFR)	0.151	0.849	2.07		
	European (EUR)	0.462	0.538	7.40		
	American (AMR)	0.480	0.520	4.90		
	East Asian (EAS)	0.355	0.645	5.40		

	South Asian (SAS)	0.462	0.538	1.73		
rs55964536		C	T		0.0044	0.9004
	African (AFR)	0.899	0.101	2.07		
	European (EUR)	0.517	0.483	7.40		
	American (AMR)	0.712	0.288	4.90		
	East Asian (EAS)	0.994	0.006	5.40		
	South Asian (SAS)	0.602	0.398	1.73		
rs734056		C	A		0.0257	0.7615
	African (AFR)	0.787	0.213	2.07		
	European (EUR)	0.511	0.489	7.40		
	American (AMR)	0.683	0.317	4.90		
	East Asian (EAS)	0.992	0.008	5.40		
	South Asian (SAS)	0.566	0.434	1.73		
rs4290734		A	G		0.0002	0.9769
	African (AFR)	0.931	0.069	2.07		
	European (EUR)	0.513	0.487	7.40		
	American (AMR)	0.689	0.311	4.90		
	East Asian (EAS)	0.993	0.007	5.40		
	South Asian (SAS)	0.569	0.431	1.73		
rs34783969		A	T		0.0066	0.8786
	African (AFR)	0.885	0.115	2.07		
	European (EUR)	0.512	0.488	7.40		
	American (AMR)	0.683	0.317	4.90		
	East Asian (EAS)	0.993	0.007	5.40		
	South Asian (SAS)	0.572	0.428	1.73		
rs11702475		C	T		0.007	0.8752
	African (AFR)	0.877	0.123	2.07		
	European (EUR)	0.509	0.491	7.40		
	American (AMR)	0.690	0.310	4.90		
	East Asian (EAS)	0.992	0.008	5.40		
	South Asian (SAS)	0.573	0.427	1.73		
rs35899679		C	A		0.0048	0.8959

		African (AFR)	0.893	0.107	2.07		
		European (EUR)	0.537	0.463	7.40		
		American (AMR)	0.712	0.288	4.90		
		East Asian (EAS)	0.993	0.007	5.40		
		South Asian (SAS)	0.616	0.384	1.73		
	rs35041537		C	T		0.0061	0.8831
		African (AFR)	0.886	0.114	2.07		
		European (EUR)	0.537	0.463	7.40		
		American (AMR)	0.709	0.291	4.90		
		East Asian (EAS)	0.993	0.007	5.40		
		South Asian (SAS)	0.615	0.385	1.73		
IL6	rs1800795		C	G		0.0136	0.8260
		African (AFR)	0.018	0.982	2.07		
		European (EUR)	0.416	0.584	7.40		
		American (AMR)	0.184	0.816	4.90		
		East Asian (EAS)	0.001	0.999	5.40		
		South Asian (SAS)	0.139	0.861	1.73		
LZTFL1	rs11385942		AA	AAA		0.1691	0.4180
		African (AFR)	0.947	0.053	2.07		
		European (EUR)	0.919	0.081	7.40		
		American (AMR)	0.954	0.046	4.90		
		East Asian (EAS)	0.995	0.005	5.40		
		South Asian (SAS)	0.704	0.296	1.73		
ABO	rs657152		A	C		0.1016	0.5380
		African (AFR)	0.461	0.539	2.07		
		European (EUR)	0.400	0.600	7.40		
		American (AMR)	0.251	0.749	4.90		
		East Asian (EAS)	0.372	0.628	5.40		
		South Asian (SAS)	0.404	0.596	1.73		
